# Editorial: Translatable Models and MRI Methods for Neurodegenerative Diseases

**DOI:** 10.3389/fnins.2022.919860

**Published:** 2022-05-05

**Authors:** Rodolfo G. Gatto, Ileana O. Jelescu, Viktor Vegh

**Affiliations:** ^1^Department of Bioengineering, University of Illinois at Chicago, Chicago, IL, United States; ^2^Department of Diagnostic Radiology and Interventional Radiology, Lausanne University Hospital (CHUV), Lausanne, Switzerland; ^3^Centre for Advanced Imaging, University of Queensland, Brisbane, QLD, Australia; ^4^ARC Training Centre for Innovation in Biomedical Imaging Technology (CIBIT), Brisbane, QLD, Australia

**Keywords:** translational models, neuroimaging, biomedical technologies and medical applications, neurodegenerative diseases, MRI

The global phenomenon of increased life expectancies in humans is attributable to advances in medical care. The incidence of neurodegenerative diseases (NDDs) goes hand-in-hand with having a larger aging population. Non-invasive imaging techniques provide opportunities to monitor the progression of NDDs and assess treatment response. The development of novel methodologies, especially those over the past few decades, lead to new knowledge and more insightful measurements of brain changes. In particular, medical imaging techniques have become forerunners in translation and routine adoption for clinical practice. In this Research Topic, we explore new insights brought by technological advances such as high-density EEG recordings, novel biomarkers derived from relaxometry and diffusion MRI, AI-powered image analysis for microbleed detection, and translatability of animal models of brain and spine injury.

Li et al. examined how dense EEG recordings in a resting state can be used to study brain dynamics in Parkinson's disease. They found movements disorders in Parkinson's disease to associate with longer resting-state microstates of the brain. They additionally showed microstate changes to be partially reversible using deep brain stimulation (DBS). Their findings promote the use of acute DBS in patients suffering from Parkinson's disease, in conjunction with a neuroimaging measure useful for patient screening and monitoring.

There is continued interest in developing MRI and specific diffusion MRI (dMRI) measures related to tissue features affected by diseases, such as primary progressive multiple sclerosis (PPMS). In a cross-sectional study, Filip et al. investigated changes in conventional (T1, T2) and adiabatic (T1ρ, T2 ρ) free precession relaxation parameters, in addition to existing dMRI derived metrics (diffusion tensor and kurtosis) to identify tissue changes. T1ρ, a marker of neuronal loss, was found to change with PPMS across numerous brain regions. Iron accumulation inferred by T2ρ maps corresponded with regions identified using T1ρ, however to a lesser extent. These modish markers of tissue changes may, through T1ρ, reflect the loss of neuronal density and its relation to iron accumulation when studied together with T2ρ. Such a technique may find application more widely, since neuronal loss and iron, particularly accumulation, are of potential interest in a multitude of NDDs.

dMRI is among the foremost innovative imaging techniques, capable of providing distinct information on neuronal tissue microstructure and composition (Gatto et al., 2018). As such, tissue-specific metrics derived from dMRI continue to prove to be of high interest to the neuroimaging community. On one hand, models have been developed based on knowledge of biology to enable direct interpretation of diffusion metrics (e.g., intra- and extra-cellular volume fraction, axon diameter, neurite density). Analytical models from the anomalous diffusion perspective have been proposed on the other hand. The interpretation of the anomalous diffusion model parameters, while classifying diffusion such as super- and sub-diffusion, has provided new opportunities for the community. In a specifically designed spinal cord study, Caporale et al. investigated the meaning of two anomalous diffusion parameters, namely α in the case of sub-diffusion and γ in super-pseudo diffusion. Interestingly, they were able to link α and local tissue heterogeneity, and γ and axon diameter and their local effective density, with the aid of histology. These findings may provide new avenues for NDDs studies since estimations of α and γ appear quite robust.

While vascular microbleeds are increasingly present with aging, they can also be insightful in vascular pathologies associated with NDDs. Concerning this, a key problem has been the automated detection of vascular microbleeds from MRI. Momeni et al. investigated the use of deep learning to detect and classify vascular microbleeds. They showed that using well-designed training data, in the form of 3D synthetic cerebral microbleed MRI patches, robust microbleed detection can be achieved even on new data previously unseen, and even on data obtained using different MRI data acquisition parameters resulting in different microbleed contrast. These findings make this deep learning tool involving the generative adversarial network potentially useful in small and large scale NDDs studies, especially when multiple sites with different protocols have been used for MRI data collection.

Neurodegeneration is a post-incident hallmark of traumatic brain injury (TBI). A key question in the field is the translatability of pre-clinical models to humans, particularly animal models which are based on mice and rats (Hutchinson et al.). The reason is that white matter and gray matter layout, particularly folding, are not present in rodent models. Increasing evidence suggests that gray-white matter organization plays an important role in the impact of TBI (Soni et al., 2020). Therefore, Hutchinson et al. used ferrets, which have gray matter folds and extensive white matter connectivity. Such a model provides new opportunities in studying damage due to TBI over the brain, as it is increasingly evident that elastic properties of tissue and its layout play a major role in the extent of the damage due to a localized insult. Hutchinson et al.'s findings on diffusion and white matter anisotropy changes with hemorrhage and edema appearing around the brain stem region are consistent with post-TBI changes leading to an extensive neurodegenerative brain disease.

Similarly, the specific clinical translation of spinal cord injury (SCI) rodent models has challenged researchers. Xing et al. focused on lesion areas and their relation to the functional performance of rats. They concluded that the smaller the lesion area the better the functional performance and behavioral outcome for the rat. Lesion area was shown to correlate as well with myelin damage as measured using T2-weighted MRI. It appears behavioral testing in conjunction with T2-weighted MRI identified changes can be used to make accurate prognostic predictions in SCI.

While neuroimaging is a mature field, there still appear to be ample opportunities to develop new, potentially translatable methods to further increase our knowledge of NDDs, as evidenced by this Research Topic.

## Author Contributions

All authors listed have made a substantial, direct, and intellectual contribution to the work and approved it for publication.

## Funding

IJ is supported by the Swiss National Science Foundation Eccellenza Fellowship PCEFP2_194260.

## Conflict of Interest

The authors declare that the research was conducted in the absence of any commercial or financial relationships that could be construed as a potential conflict of interest.

## Publisher's Note

All claims expressed in this article are solely those of the authors and do not necessarily represent those of their affiliated organizations, or those of the publisher, the editors and the reviewers. Any product that may be evaluated in this article, or claim that may be made by its manufacturer, is not guaranteed or endorsed by the publisher.
